# Assessing caregiver stress and resource needs in pediatric cancer care

**DOI:** 10.1186/s12912-024-02483-x

**Published:** 2024-12-18

**Authors:** Shaimaa Mohamed Amin, Mahmoud Abdelwahab Khedr, Azza Medhat Aziz Mansy, Ahmed Hashem El-Monshed, Mohamed Gamal Noaman Malek, Ayman Mohamed El-Ashry

**Affiliations:** 1 Community Health Nursing, Faculty of Nursing, Damanhour University, Damanhour city, Egypt; 2 Psychiatric and Mental Health Nursing, Faculty of Nursing, Alexandria University, Alexandria, Egypt; 3 Department of Nursing, College of Applied Medical Sciences, Hafr Albatin University, Hafr Albatin, Saudi Arabia; 4 Psychiatric and mental health nursing faculty of nursing, Damanhour University, Damanhour, Egypt; 5 Department of Psychiatric and Mental Health Nursing, Faculty of Nursing-Mansoura University, Mansoura, Egypt; 6 Department of Nursing, College of Health and Sport Sciences, University of Bahrain, Manama, Bahrain; 7 Community Health Nursing, Faculty of Nursing, Minia University, Minia, Egypt; 8 Psychiatric and Mental Health Nursing, Department of Nursing, College of Applied Medical Sciences, Jouf University, Al Qurayyat, Saudi Arabia

**Keywords:** Caregiver stress, Pediatric cancer, Resource needs, Oncology care

## Abstract

**Background:**

Caregivers of pediatric cancer patients often face significant stress and emotional strain, which can negatively impact their well-being and the quality of care provided to their children. Understanding the socio-demographic factors influencing caregiver stress and identifying the specific needs and resources required by caregivers are crucial for improving support systems in pediatric cancer care.

**Aim of the study:**

This study aimed to analyze the levels of caregiver stress and their resource needs within pediatric cancer care.

**Methods:**

A cross-sectional descriptive design was used, and the study was conducted at El-Minia Oncology Center outpatient clinics, Egypt. The sample included 258 pediatric cancer caregivers selected based on predefined eligibility criteria. Data were collected using the Caregiver Needs and Resources Assessment (CNRA) and the King Stone Caregiver Stress Scale (KCSS), both of which demonstrated strong reliability and validity. Descriptive statistics, t-tests, ANOVA, and Pearson correlation coefficients were employed for data analysis using SPSS version 29, with a significance level set at *p* < 0.05.

**Results:**

Caregivers reported moderate to high stress levels, with an average stress score of 34.59 (SD = 7.649). Age, education level, marital status, and income were significant predictors of caregiver stress (*p* < 0.001), with lower income and higher educational levels contributing to increased stress. The number of children, child’s age, and duration since cancer diagnosis also significantly impacted stress levels (*p* < 0.001). Correlation analysis revealed that psychological needs were positively related to stress (*r* = 0.488, *p* < 0.001), while spirituality was negatively associated with stress (*r* = -0.241, *p* < 0.001). Regression analysis indicated that physiological needs (ß = 0.331) and role conflict (ß = 0.294) were the strongest predictors of stress (R² = 0.636).

**Conclusion and implications:**

This study emphasizes the impact of socio-demographic factors on caregiver stress in pediatric cancer care. Targeted interventions that address caregivers’ psychological, social, and financial needs are essential to alleviate stress. Implementing caregiver-centered support programs in pediatric oncology can enhance the well-being of both caregivers and patients. Future research should explore sustainable strategies to further reduce caregiver burden.

**Clinical trial number:**

Not applicable.

## Introduction

Cancer continues to stand as one of the most formidable and harrowing diagnoses that any family might ever confront, with the ramifications of such a life-altering diagnosis becoming even more severe and emotionally devastating when it pertains to a child, who is inherently more vulnerable and dependent. Pediatric cancer, while statistically occurring less frequently than its adult counterpart, introduces a distinctive array of complications and hurdles that extend far beyond the mere medical treatment and clinical oversight of the affected young patient, thereby necessitating a comprehensive approach that encompasses various aspects of care [1].

These challenges require a profound and nuanced understanding of the psychosocial dimensions that are inextricably linked to the experience of illness, not only for the child who is enduring the trauma but also for their caregivers, most notably the parents, who assume an irreplaceable and critical role in guiding the child through the arduous journey of battling cancer and its associated treatments [2].

Pediatric cancer represents a significant public health issue that profoundly alters the quality of life not only for the children who receive a cancer diagnosis but also for their families, who often bear the emotional and logistical burdens accompanying such a diagnosis. In the year 2019 alone, it was estimated that approximately 1.8 million children were living with cancer, and concurrently, 291,319 new cases were reported, indicating a troubling trend that requires urgent attention [3]. The burden of pediatric cancer is particularly pronounced in low- and middle-income countries (LMICs), which are responsible for an overwhelming 90% of global pediatric cancer cases, highlighting a significant disparity in health outcomes and access to treatment resources. This stark contrast underscores the alarming differences in treatment success rates, with high-income countries achieving cure rates that exceed 80%, whereas the rates in LMICs often plummet below 30%, primarily due to systemic differences in healthcare access, resources, and infrastructure [4, 5].

Such statistics serve to illuminate the essential role that family caregivers play in the care continuum, as they often find themselves at the epicenter of managing the complex and multifaceted needs of a child diagnosed with cancer, navigating a landscape fraught with emotional and logistical challenges.

Numerous studies have illustrated that the effects of childhood cancer on parents can frequently be interpreted through the prism of trauma-related symptoms, which are commonly referred to as posttraumatic stress symptoms (PTSS), further complicating their emotional landscape and coping strategies [6, 7]. According to Norberg et al. (2005), the adjustment process emerges as a pivotal phase in the overall psychological stress response, serving as a critical turning point for parents as they strive to navigate the overwhelming complexities associated with their child’s illness, ultimately shaping their coping mechanisms and emotional resilience in the face of adversity [8].

Upon receiving the heartrending and devastating news regarding their child’s diagnosis of cancer, parents are abruptly thrust into an overwhelming and tumultuous journey that is marked by an array of significant psychological stressors that are often difficult to comprehend and navigate [8]. The stress response experienced by these caregivers is intricate and multifaceted, typically manifesting as profound emotional strain that may encompass symptoms of anxiety, depression, and situational turmoil characterized by feelings of uncertainty, helplessness, loneliness, and acute fears regarding the future and the implications of their child’s illness [8, 9].

Lazarus and Springer’s (1984) stress and coping theory provides a robust framework for understanding the complex psychological processes that occur when parents are confronted with their child’s cancer diagnosis. This theory suggests that parents initially assess the stressor, followed by an evaluation of the strain—conceptualized as the pressures or demands they face—leading to stress responses such as uncertainty, pervasive worry, depressive symptoms, and the emergence of PTSS [10].

Beyond the psychological toll, caregivers of pediatric cancer patients often face significant unmet needs across several domains, which can adversely affect patient outcomes. Emotional support, logistical assistance, and access to information regarding diagnosis and treatment are critical areas of need. For instance, 58.8% of caregivers report emotional concerns such as fear and anxiety, while logistical needs for managing daily activities and understanding treatment side effects are also prevalent [11]. The strain on caregivers extends to somatic, economic, and psychosocial challenges, which can diminish their well-being and, in turn, the quality of care they provide to their children [12].

Though a potential resource for information and social support, the internet needs to be more utilized by many caregivers, with only 43% discussing the information they find with healthcare providers [13]. This gap underscores the need for structured support systems, such as caregiver support groups, to address these unmet needs and enhance the caregiving experience [11]. By optimizing supportive care through targeted interventions, we can improve not only patient outcomes but also the resilience and well-being of caregivers [14].

### Significance of the study

Despite the considerable volume of scholarly investigation that has been conducted regarding the psychosocial repercussions experienced by caregivers of pediatric cancer patients, there continues to exist a multitude of significant deficiencies in our comprehensive understanding of the entire range of their diverse needs, as well as the most efficacious methodologies for providing them with appropriate support and assistance. Current academic studies frequently concentrate on the immediate emotional distress and logistical obstacles these caregivers encounter. Furthermore, the stark and notable disparities in healthcare access and availability between high-income nations and low- to middle-income countries highlight the critical importance of developing and implementing tailored interventions that specifically address the unique challenges caregivers face in particular regional contexts. This study is designed with the explicit purpose of bridging these identified gaps in the existing literature by offering a thorough and comprehensive evaluation of caregiver stress levels and resource requirements, with the ultimate goal of contributing to the formulation of more effective support systems that have the potential to significantly enhance the overall well-being and quality of life for both caregivers themselves as well as the pediatric cancer patients for whom they provide care.

### Aim of the study

The aim of this study is to investigate the levels of stress experienced by caregivers of pediatric cancer patients and to assess the adequacy and accessibility of resources available to them. Through a community nursing-led design, the study seeks to identify gaps in support systems and develop strategies to enhance caregiver well-being and resource utilization in pediatric cancer care settings.

## Methods

### Study design and setting

This study used a cross-sectional descriptive research design, adhering to the Strengthening the Reporting of Observational Studies in Epidemiology (STROBE) guidelines. The study was conducted at El-Minia oncology center outpatient clinics, Egypt. As a comprehensive oncology facility, it offers diagnostic, therapeutic, and supportive services tailored to the unique needs of pediatric patients. This includes specialized treatments such as chemotherapy, radiotherapy, surgery, and supportive care aimed at managing the physical, emotional, and psychological aspects of cancer in children.

### Sample size and study population

The target group for this study was pediatric cancer caregivers. To be eligible, participants had to be primary caregivers of children less than 18 years old diagnosed with cancer. Exclusion criteria focused on specific circumstances that could affect the caregiving experience. Individuals were excluded if they had provided care for less than three months, ensuring that the study captured more stable caregiving experiences. Additionally, those with severe physical or mental health conditions that could independently contribute to high stress levels were excluded to prevent confounding results.

The sample size for this study was determined based on data from a previous cross-sectional study that measured caregivers’ stressful care experiences [15]. The study reported a mean score of 20.2 with a standard deviation of 7.3. Using a 95% confidence level and a margin of error of 5%, the sample size was estimated at 200 participants, calculated with the formula [16] n = (Z * σ / E)². Where Z is 1.96 (for 95% confidence), σ is 7.3, and E is 5% of the mean (1.01). To account for potential dropouts and non-responses, an adjustment of 30% was applied [17], resulting in a final target sample size of 278 caregivers. After considering participants who did not meet the inclusion criteria and those who declined to participate, the final sample size was 258 caregivers.

## Measurements of interests

### The demographic form

The demographic form collected data about the primary caregiver and the child, including caregiver role (mother, grandmother, aunt), age (20–<30 years, 30–<40 years, ≥ 40 years), education level (illiterate, basic education, secondary education, university & post-graduate), marital status (married, divorced/widowed), residence (rural, urban), employment status (working, not working), and family income (enough, not enough, enough & save). Additionally, data were gathered on the number of children in the caregiver’s family (1–2, 3–4, ≥ 5), and information regarding the child with cancer, such as age (< 10 years, ≥ 10 years), gender (male, female), duration since cancer diagnosis (< 2 years, 2–<4 years, ≥ 4 years), and birth order (first, second, third, fourth). These sociodemographic groupings were used to analyze their impact on caregiver stress and resource needs. In addition to the caregiver data collected, the study also gathered information regarding the type of cancer diagnosis and the phase of illness. These factors are crucial, as they significantly influence the caregiving experience, and the resources and skill sets required during different phases of the illness.

### The caregiver needs and resources assessment (CNRA)

The Caregiver Needs and Resources Assessment (CNRA) was developed by Li et al., 2023 [18] to evaluate the needs and resources of caregivers. The tool comprises 36 items across two dimensions. The needs dimension includes physiological needs (3 items), role conflict (3 items), care recipient’s needs (3 items), psychological needs (3 items), and social support needs (3 items). The resources dimension consists of spirituality (4 items), self-efficacy (3 items), responsibility and commitment as a caregiver (3 items), community resources (2 items), family support (3 items), healthy lifestyle (3 items), and closeness with the care recipient (3 items). Items 31 and 32 are reverse-scored. Each item is rated on a five-point Likert scale ranging from 1 (never) to 5 (very often). A higher total score indicates greater unmet needs and insufficient resources for the caregiver. According to Li et al., 2023, the scale demonstrated an excellent psychometric properties. The CNRA subscales demonstrated good reliability, with Cronbach’s alpha ranging from 0.705 to 0.888. The overall reliability of the tool was confirmed with a coefficient of *r* = 0.892, indicating a high level of consistency. Exploratory factor analysis was conducted to assess the instrument’s validity. The factor loadings ranged from 0.442 to 0.829 before rotation and improved to 0.580 to 0.953 after varimax rotation, all exceeding the threshold of 0.35. These factors collectively accounted for 68.606% of the total variance, supporting the validity of the CNRA.

In the present study, the overall reliability of the CNRA was confirmed with a coefficient of *r* = 0.912, indicating a high level of internal consistency. To assess the validity of the CNRA, exploratory factor analysis was conducted within our study sample. The factor loadings ranged from 0.552 to 0.882 prior to rotation and improved to 0.620 to 0.980 after varimax rotation, with all loadings exceeding the threshold of 0.35. These factors collectively accounted for 71.342% of the total variance, supporting the validity of the CNRA for our specific population of pediatric cancer caregivers.

### King Stone Caregiver stress scale (KCSS)

This scale, developed by Hopkins et al., 2015 [19], is designed to allow caregivers to quickly indicate their stress levels. It comprises ten questions, each addressing different potential sources of caregiver stress, such as emotional strain, family dynamics, and financial issues. Respondents rate their stress for each item on a scale from [1] no stress to [5] extreme stress. Higher scores indicate greater levels of stress. According to Hopkins et al., 2015, the scale exhibited outstanding psychometric properties. Reliability testing showed the tool is reliable, with a reliability coefficient of *r* = 0.981. Additionally, an exploratory factor analysis was performed to evaluate the scale’s validity. Factor loadings ranged from 0.612 to 0.715 before rotation and improved to between 0.552 and 0.878 after varimax rotation, all exceeding the threshold of 0.35. These factors accounted for 67.547% of the total variance. Moreover, the Kaiser-Meyer-Olkin measure of sampling adequacy was 0.885, suggesting the data were highly suitable for factor analysis.

In our study, the overall reliability of the scale was confirmed with a coefficient of *r* = 0.925, indicating a high level of internal consistency. To assess the validity of the scale, exploratory factor analysis was conducted within our study sample. The factor loadings ranged from 0.480 to 0.850 prior to rotation and improved to 0.610 to 0.970 after varimax rotation, with all loadings exceeding the threshold of 0.35. These factors collectively accounted for 72.150% of the total variance, supporting the validity of the scale for our specific population of pediatric cancer caregivers.

### Study procedures

#### Tool preparation &Pilot study

The translation of the research instruments, including CNRA and KCSS, into Arabic was undertaken with meticulous care. Bilingual experts fluent in both English and Arabic ensured accurate and culturally appropriate translations. These translations were then back-translated into English to confirm linguistic equivalence and resolve any discrepancies. After translation, face validity assessments were conducted for each instrument. Permission to translate the tools was obtained from the original publisher. Expert panels in the field of community health nursing and psychiatric and mental health nursing reviewed the translated tools to ensure they effectively captured the intended constructs in the Arabic context. Feedback from potential participants was also gathered to confirm the clarity, relevance, and cultural appropriateness of the translated items. Subsequently, reliability measures were implemented. Internal consistency reliability was assessed using statistical methods such as Cronbach’s alpha to ensure consistency within each scale. Regarding the pilot study, it involved a separate group of 25 caregivers who were not part of the final sample of 258 study participants. The pilot study was conducted to evaluate the clarity, relevance, and reliability of the study tools before the main data collection. The findings indicated that the instruments were effective, and no modifications were necessary.

### Data collection

The data collection process began with the researcher providing participants with a thorough orientation that outlined the study’s objectives and emphasized the voluntary nature of participation. All questions and concerns from participants were addressed promptly, and confidentiality measures were clearly communicated to establish trust. Data collection took place from early July 2024 to late August 2024. Participation required both oral and written informed consent. To accommodate participants who were illiterate, the researcher conducted interviews with caregivers, guiding them through the structured questionnaires in the outpatient clinic’s waiting area. Each session lasted between 30 and 35 min and took place five days a week, from 9:00 AM to 2:00 PM. This approach ensured that all caregivers could participate meaningfully in the study, regardless of their literacy levels.

### Ethical considerations

Approval was obtained from the Research Ethics Committee of the Faculty of Nursing, Minia University in Egypt (Code; REC202474). The rights and safety of the participants were protected in accordance with local laws, regulations, and the ethical principles outlined in the Declaration of Helsinki. Participants were provided with clear information regarding the study’s objectives and were explicitly informed that their participation was entirely voluntary and anonymous. They were assured that all collected data would be handled with the utmost confidentiality, accessible only to authorized members of the research team. Written informed consent was obtained from each participant before data collection began. Participants were also informed that they could withdraw from the study at any time without any consequences.

### Statistical analysis

Version 29 of SPSS was employed to conduct comprehensive analyses of the collected data. Descriptive statistics, including percentages and frequency counts, were used to summarize categorical variables such as sociodemographic factors of both the child and the caregiver. Means and standard deviations were calculated for continuous variables like caregiver stress and needs scores. To compare caregiver stress and needs across sociodemographic factors, Student’s t-tests were used for binary comparisons (e.g., rural vs. urban caregivers, marital status) and one-way Analysis of Variance (ANOVA) was conducted to assess differences across multiple categories (e.g., different education levels and age groups). Cohen’s d and eta-squared (η²) were used to report effect sizes, highlighting the practical significance of these differences. Pearson correlation coefficients (r) were calculated to explore associations between continuous variables, such as the relationship between caregiver stress and psychological needs, spirituality, self-efficacy, and other caregiver resources. A multiple regression analysis was performed to identify significant predictors of caregiver stress, with variables such as psychological needs, physiological needs, role conflict, healthy lifestyle, and community resources being assessed for their contribution to the overall stress experienced by caregivers. All statistical tests were performed with the aim of identifying significant relationships and differences in caregiver stress and needs, adhering to the alpha level of 0.05 to determine statistical significance.

## Results

### Socio-demographic characteristics and significant relationships

As shown in Table 1, the majority of caregivers were mothers, comprising 92.6% of the sample. The age distribution of caregivers predominantly featured those aged between 30 and 40 years, accounting for 60.1% of the group. Among the caregivers, 55.0% had attained secondary education. The residential status of caregivers indicated that 57.8% lived in urban areas. More than half of the caregivers, at 53.5%, were not employed. Most of the children diagnosed with cancer were under ten years old, comprising 64.0% of the sample. The gender distribution showed that 60.5% of the affected children were female.

**Table 1:** Socio-demographic characteristics of the study participants (*N* = 258)

Socio-demographic Factors			*N*	*%*	
Child Primary Caregiver		Mother	239	92.6	
		Grandmother	13	5.0	
		Aunt	6	2.3	
Caregiver’s Age (in Years)		20 - (> 30)	81	31.4	
		30 - (> 40)	155	60.1	
		≥ 40	22	8.5	
Caregiver’s Level of Education		Illiterate	31	12.0	
		Basic Education	57	22.1	
		Secondary Education	142	55.0	
		University & post-graduate	28	10.9	
Caregiver’s Marital Status		Married	216	83.7	
		Divorced/ Widowed	42	16.3	
Caregiver’s Residence		Rural	109	42.2	
		Urban	149	57.8	
Caregiver’s Employment Status		Working	120	46.5	
		Not Working	138	53.5	
Caregiver’s Family Income		Enough	178	69.0	
		Not Enough	43	16.7	
		Enough & save	37	14.3	
Number of Children in the Family of the Caregiver		1–2	104	40.3	
		3–4	145	56.2	
		≥ 5	9	3.5	
Age of the Child with Cancer (in Years)		< 10	165	64.0	
		≥ 10	93	36.0	
Gender of the Child with Cancer	Male	102		39.5	
	Female	156		60.5	
Duration of Cancer Diagnosis (in Years)	< 2	110		42.6	
	2 - (> 4)	91		35.3	
	≥ 4	57		22.1	
Birth order of the Child with Cancer	First	70		27.1	
	Second	130		50.4	
	Third	50		19.4	
	Fourth	8		3.1	

### Descriptive statistics of study measures and their domains

As shown in Table 2, the overall mean caregiver stress score was 34.59 (SD = 7.649), with scores ranging from 15 to 50. The domain scores revealed that caregiving issues (M = 23.98, SD = 5.421) contributed the most to the total stress, followed by family issues (M = 6.86, SD = 1.972), and financial issues (M = 3.74, SD = 1.019). For caregiver needs and resources, the total mean score was 115.19 (SD = 10.238), with a range from 85 to 152. Among the domains, spirituality had the highest mean score (M = 12.82, SD = 3.027), followed by psychological needs (M = 11.31, SD = 2.510) and social support needs (M = 11.90, SD = 2.590). The lowest mean score was observed for community resources (M = 4.31, SD = 1.772).

**Table 2:** Descriptive statistics of the study measures (*N* = 258)

Study Measures	M	SD	Range	Minimum	Maximum
Caregiver Stress (Total)	34.59	7.649	35	15	50
• Caregiving Issues	23.98	5.421	24	11	35
• Family Issues	6.86	1.972	8	2	10
• Financial Issues	3.74	1.019	4	1	5
**Caregiver Needs and Resources (Total)**	**115.19**	**10.238**	**67**	**85**	**152**
• Physiological Needs	11.28	2.427	9	6	15
• Role Conflict	9.92	2.845	11	4	15
• Care Recipient’s Needs	10.15	2.516	11	4	15
• Psychological Needs	11.31	2.510	9	6	15
• Social Support Needs	11.90	2.590	11	4	15
• Spirituality	12.82	3.027	16	4	20
• Self-Efficacy	8.50	2.415	12	3	15
• Responsibility and Commitment	9.33	2.179	11	4	15
• Community Resources	4.31	1.772	8	2	10
• Family Support	8.81	2.310	12	3	15
• Closeness with the Care Recipient	10.43	1.189	8	6	14
• Healthy Lifestyle	6.45	2.354	10	3	13

M = Mean, SD = Standard Deviation

### Correlation analysis between study measures

Table 3 highlights key relationships. Psychological Needs showed a positive correlation with caregiver stress (*r* = 0.488, *p* < 0.001). In contrast, Spirituality was negatively correlated with caregiver stress (*r* = -0.241, *p* < 0.001). Self-Efficacy also had a negative correlation with stress (*r* = -0.326, *p* < 0.001). Additionally, a Healthy Lifestyle was strongly negatively correlated with stress (*r* = -0.490, *p* < 0.001).

**Table 3:** Correlation analysis between the study measures (*N* = 258)

Study Measures	Caregiving Issues	Family Issues	Financial Issues	Caregiver Stress (Total)
Physiological Needs	0.676^**^	0.542^**^	0.529^**^	0.690^**^
Role Conflict	0.626^**^	0.541^**^	0.422^**^	0.639^**^
Psychological Needs	0.417^**^	0.499^**^	0.477^**^	0.488^**^
Social Support Needs	0.220^**^	0.358^**^	0.391^**^	0.300^**^
Spirituality	-0.227^**^	-0.235^**^	-0.146^*^	-0.241^**^
Self-Efficacy	-0.283^**^	-0.299^**^	-0.365^**^	-0.326^**^
Community Resources	-0.059	-0.188^**^	-0.232^**^	-0.122
Family Support	-0.287^**^	-0.295^**^	-0.240^**^	-0.312^**^
Healthy Lifestyle	-0.490^**^	-0.408^**^	-0.338^**^	-0.497^**^
**Caregiver Needs and Resources (Total)**	0.178^**^	0.211^**^	0.211^**^	0.209^**^

Note: Only key correlations are highlighted for brevity

r **=** Pearson Correlation

**. Correlation is significant at the 0.01 level (2-tailed)

*. Correlation is significant at the 0.05 level (2-tailed)

### Relationship of socio-demographic factors with caregiver stress and caregiver needs and resources

Table 4 revealed several significant relationships between socio-demographic factors and caregiver stress. Caregivers aged ≥ 40 years reported higher stress compared to younger caregivers (*p* < 0.001). Caregivers holding university or post-graduate degrees reported lower stress levels (*p* < 0.001). Divorced or widowed caregivers had higher stress than married ones (*p* = 0.003), and rural caregivers reported more stress than their urban counterparts (*p* = 0.030). Additionally, caregivers with more children (*p* < 0.001) and those whose children were older (≥ 10 years, *p* < 0.001) or had been diagnosed with cancer for longer periods (≥ 4 years, *p* < 0.001) reported higher stress. For caregiver needs and resources, significant differences were found for caregiver age (*p* = 0.018), duration of the cancer diagnosis (*p* = 0.030), and birth order (*p* = 0.011).

**Table 4:** Relationship of Socio-Demographic Factors with caregiver stress and caregiver needs and resources (*N* = 258)

Socio-demographic Factors		Caregiver Stress		Caregiver Needs and Resources	
		*M*	*SD*	*M*	*SD*
Caregiver’s Age (in Years)	20 - (> 30)	31.90	8.311	112.53	12.389
	30 - (> 40)	35.72	7.044	116.40	8.835
	≥ 40	36.45	6.933	116.50	9.257
	F (*P*)	7.743 (< 0.001)**		4.091 (0.018)*	
	η2	0.057		0.031	
Caregiver’s Level of Education	Illiterate	35.19	7.993	115.06	10.478
	Basic Education	36.47	8.426	116.02	11.634
	Secondary Education	35.12	6.892	114.84	9.614
	University & post-graduate	27.36	5.194	115.46	10.493
	F (*P*)	10.923 (< 0.001)**		0.187 (0.905)	
	η2	0.114		0.002	
Caregiver’s Marital Status	Married	33.96	7.765	115.19	10.653
	Divorced/ Widowed	37.79	6.167	115.19	7.862
	t (*P*)	-3.010 (0.003)**		0.002 (0.998)	
	d	7.532		10.258	
Caregiver’s Residence	Rural	35.79	7.485	115.91	11.197
	Urban	33.70	7.673	114.67	9.479
	t (*P*)	2.178 (0.030)*		0.959 (0.339)	
	d	7.594		10.239	
Number of Children in the Family of the Caregiver	1–2	31.24	8.190	113.92	10.426
	3–4	36.63	6.484	115.95	10.153
	≥ 5	40.22	1.394	117.67	8.588
	F (*P*)	20.223 (< 0.001)**		1.466 (0.233)	
	η2	0.137		0.011	
Age of the Child with Cancer (in Years)	< 10	33.25	8.022	114.56	11.335
	≥ 10	36.96	6.312	116.31	7.859
	t (*P*)	-3.838 (< 0.001)**		-1.455 (0.147)	
	d	7.453		10.223	
Duration of Cancer Diagnosis (in Years)	< 2	32.21	8.318	115.43	10.915
	2 - (> 4)	35.36	6.378	113.27	10.542
	≥ 4	37.93	6.708	117.81	7.582
	F (*P*)	12.205 (< 0.001)**		3.554 (0.030)*	
	η2	0.087		0.027	

* Correlation is significant at the 0.05 level (2-tailed), ** Correlation is significant at the 0.01 level (2-tailed)

*M* = Mean; *SD* = Standard Deviation; Test of Significance; *t =* Student t test; *F* = ANOVA test

η^2^ = Eta-squared effect size; Small (0.01 ≤ η^2^ < 0.06), Medium (0.06 ≤ η^2^ < 0.14), Large (η^2^ ≥ 0.14)

d = Cohen’s d effect size; Small (0.01 ≤ d < 0.50), Medium (0.50 ≤ d < 0.80), Large (d ≥ 0.80)

### Regression analysis of caregiver needs and resources as predictors of caregiver stress

As shown in Table 5, the regression analysis indicates several significant predictors of caregiver stress. Physiological Needs (ß = 0.331, *p* < 0.001) and Role Conflict (ß = 0.294, *p* < 0.001) are significant positive predictors of caregiver stress. Psychological Needs (ß = 0.213, *p* < 0.001) also significantly predict higher caregiver stress. In contrast, Healthy Lifestyle (ß = -0.174, *p* < 0.001) significantly negatively predicts caregiver stress. Community Resources (ß = 0.098, *p* = 0.046) is a positive predictor of stress. Other predictors such as Spirituality and Self-Efficacy did not significantly predict caregiver stress. The model explained 63.6% of the variance in caregiver stress (R² = 0.636).

**Table 5:** Regression coefficients: Caregiver needs and resources domains as predictors of caregiver stress

Predictors	Caregiver Stress						
	Unstandardized Coefficients		Standardized Coefficients	t	*P*	95% CI for difference	
	B	Std. Error	ß			Lower bound	Upper bound
**Constant**	11.180	4.158		2.689	0.008	2.990	19.369
Physiological Needs	1.042	0.165	0.331	6.297	< 0.001	0.716	1.368
Role Conflict	0.789	0.154	0.294	5.132	< 0.001	0.486	1.092
Psychological Needs	0.650	0.164	0.213	3.973	< 0.001	0.328	0.972
Spirituality	-0.128	0.127	-0.050	-1.002	0.317	-0.378	0.123
Self-Efficacy	0.073	0.171	0.023	0.426	0.671	-0.263	0.409
Community Resources	0.421	0.210	0.098	2.006	0.046	0.008	0.835
Healthy Lifestyle	-0.564	0.161	-0.174	-3.503	< 0.001	-0.882	-0.247

*ß* = standardized coefficient beta; *t =* Student t test for linear regression

95% CI for difference = 95% Confidence Interval for Difference

*R*^*2*^ = 0.636 (*Adj R*^*2*^ = 0.618), *F-*change = 35.667, *P* < 0.001

Dependent variable: Caregiver Stress

## Discussion

Caring for a child with cancer places significant physical, emotional, and financial strain on parents and family members. The current study aimed to evaluate caregiver stress and resource needs in pediatric cancer care. The current results indicate that various socio-demographic factors influence caregiver stress, including age, education level, marital status, and financial stability. Understanding these factors is crucial for developing targeted interventions to support caregivers effectively.

The findings from this study reveal that caregiving issues emerged as the primary contributor to overall caregiver stress. This result emphasizes the considerable strain experienced by caregivers as they navigate the daily challenges of caring for a child with cancer. The elevated stress levels related to caregiving tasks align with previous studies that highlight the intricate and demanding nature of pediatric cancer care. Research has shown that caregivers often face a complex array of responsibilities, including coordinating medical treatments, managing numerous appointments, and addressing both the physical and emotional needs of the child with cancer [20].

Family issues represented the second-highest source of stress for caregivers. This result highlights the ripple effect that pediatric cancer can have on family dynamics, potentially straining relationships and disrupting family routines. The stress arising from family issues may reflect challenges in balancing the needs of the ill child with those of other family members, as well as navigating changes in family roles and responsibilities [21]. Financial issues, while scoring lower than the other domains, still contributed to overall caregiver stress. This finding is consistent with research documenting the financial strain experienced by families dealing with pediatric cancer, including out-of-pocket expenses, loss of income due to caregiving responsibilities, and the long-term financial impact of treatment costs [22].

The study reveals that caregiver age plays a critical role in stress levels, with those in the 30 to 40 age range reporting the highest stress. This aligns with existing literature suggesting that middle-aged caregivers often juggle multiple responsibilities, including work and family obligations, which can exacerbate stress [21]. Moreover, the level of education significantly impacts stress levels, as caregivers with only secondary education experience higher stress. This finding underscores the importance of educational resources and support systems that can empower caregivers with knowledge and coping strategies [23]. Marital status also emerges as a significant factor, with divorced or widowed caregivers reporting higher stress levels compared to their married counterparts. This is consistent with previous research indicating that single caregivers often lack the emotional and practical support that married caregivers may receive, leading to increased feelings of isolation and stress [22]. Additionally, the residence of caregivers—whether rural or urban—affects stress levels, with rural caregivers reporting higher stress. This may be attributed to limited access to healthcare resources and social support networks in rural areas, which can intensify the challenges of caregiving [24].

Financial stability is another critical factor influencing caregiver stress. Caregivers who reported their income as “not enough” experienced significantly higher stress levels. This finding highlights the financial strain that often accompanies caregiving for a child with cancer, where medical expenses can be overwhelming [25]. The study suggests that improving financial support for caregivers could alleviate some of the stress associated with their caregiving responsibilities. The number of children in the family also correlates with caregiver stress, with those caring for multiple children reporting higher levels of stress. This may be due to the increased demands on time and resources when managing the care of several children, particularly when one is undergoing cancer treatment [20].

In terms of caregiver needs and resources, the high scores in the spirituality, psychological needs, and social support domains underscore the importance of providing holistic support to this population. Spiritual coping mechanisms have been shown to help caregivers find meaning and resilience in the face of adversity [26]. Addressing psychological needs, such as anxiety and depression, is crucial for maintaining caregiver mental health [20]. Social support from family and the broader community can buffer the negative effects of caregiving stress and promote overall adjustment [27]. The relatively low score for community resources suggests that more efforts are needed to ensure caregivers can access practical support, such as respite care, support groups, and financial assistance. Lack of community resources can exacerbate the isolation and burden experienced by caregivers [28].

The correlation analysis reveals several important relationships between caregiver stress and various factors related to needs, resources, and coping mechanisms. The positive correlation between psychological needs and caregiving issues, family issues, and overall stress highlights the significant emotional toll experienced by caregivers. Caring for a child with cancer can be psychologically demanding, leading to feelings of anxiety, depression, and burnout [20]. Moreover, the negative correlation between spirituality and stress suggests that engaging in spiritual practices or finding meaning in one’s circumstances can serve as a protective factor against the detrimental effects of caregiving stress. Spirituality can provide a sense of purpose, comfort, and resilience in adversity. Encouraging caregivers to explore their spiritual beliefs and practices may be a valuable strategy in stress management [26].

In the Egyptian context, spirituality often serving as a source of strength and resilience for caregivers. Many individuals find comfort in religious practices and community engagement, which can help mitigate feelings of isolation and anxiety [29]. Engaging in spiritual coping mechanisms can offer emotional relief and strengthen community bonds as caregivers share experiences and support one another. Moreover, family structures in Egypt also play a crucial role in caregiver experiences. The traditional emphasis on family support can be both a source of strength and a source of stress. Caregivers often rely on extended family members for emotional and practical support, which can alleviate some of the burdens associated with caregiving [30]. However, the study shows that the lack of adequate community resources can exacerbate stress levels, indicating that while family support is significant, it may not always be sufficient to meet the diverse needs of caregivers.

Self-efficacy, or one’s belief in their ability to handle challenging situations, is inversely related to caregiving stress. Caregivers who feel more confident in their skills and abilities may be better equipped to manage the demands of caregiving, leading to reduced stress levels. Providing caregivers with training, education, and support to enhance their self-efficacy could effectively mitigate stress [20]. Furthermore, the negative correlation between family support and stress underscores the importance of having a strong support system for caregivers. When caregivers feel supported by their family members, they may experience less stress and burden [31]. In addition, the strong negative correlation between a healthy lifestyle and stress indicates that engaging in self-care activities, such as exercise, proper nutrition, and stress management techniques, can significantly reduce caregiver stress. Caregivers who prioritize their own well-being may be better equipped to handle the demands of caregiving [32].

Moreover, the regression analysis highlights the complex interplay of factors contributing to caregiver stress, identifying physiological needs, role conflict, psychological needs, and healthy lifestyle habits as significant predictors. Caregivers’ basic physiological needs, such as sleep, nutrition, and overall health, must be met to reduce stress levels, as unmet needs can lead to heightened stress. Similarly, role conflict, often arising from juggling multiple responsibilities, contributes to increased stress. Conversely, engaging in a healthy lifestyle, including self-care practices like regular exercise, balanced nutrition, and stress management techniques, can protect against stress. While access to community resources is beneficial, it may not be sufficient to significantly alleviate stress, and caregivers may require additional support in navigating and utilizing these resources effectively. These findings align with existing literature emphasizing the importance of fulfilling caregivers’ basic needs, reducing conflicting demands, enhancing their psychological well-being, and promoting healthy lifestyle choices to improve their overall well-being [20, 32, 33].

### Limitations of the study

Several limitations should be acknowledged in this study. Firstly, the sample was limited to El-Minia oncology center outpatient clinics, in Egypt, which may affect the generalizability of the findings to other populations or regions. Additionally, the study’s cross-sectional design precludes any causal inferences between care giver stress and issues and caregiver needs and resources. Because participants may give socially acceptable replies, response biases could be introduced by relying solely on self-reported data. Furthermore, the study did not account for potential confounding variables such as the source of social support, extended or nuclear family, care givers hobbies, their perception toward own general health and chronic illness or disability that represent additional stress, burden and commitment and affect their pattern of coping. Future research should consider longitudinal designs, diverse populations, and additional contextual factors to build on these findings.

### Implications for nursing practice and policy

The findings from this study have several important implications for nursing practice, education, and healthcare policy. First, the study underscores the importance of providing targeted support to caregivers based on their specific socio-demographic characteristics. Tailored interventions that address the unique stressors of mothers, older caregivers, and those with lower education levels or financial instability should be prioritized in pediatric cancer care settings. Nurses and healthcare teams should focus on assessing the psychological and emotional well-being of caregivers, as well as ensuring they have access to necessary resources, including spiritual and social support.

From a policy perspective, the results suggest the need for healthcare systems to implement caregiver support programs that extend beyond medical care. These programs should include mental health services, financial counseling, and community-based resources designed to alleviate stress and address caregivers’ needs. Additionally, training programs for nurses should emphasize the importance of caregiver support and equip healthcare professionals with the skills to identify and mitigate stress factors effectively. This will not only improve caregiver well-being but also enhance the overall care experience for pediatric cancer patients and their families.

## Conclusion

This study provides significant insights into the factors influencing caregiver stress and resource needs in pediatric cancer care. The results indicate that socio-demographic factors, such as age, education level, marital status, and residence, play a crucial role in determining stress levels among caregivers. Additionally, factors such as family size, the child’s age, duration of the cancer diagnosis, and birth order were found to significantly affect caregiver stress. The findings also highlight that financial stability, a caregiver’s self-efficacy, and access to spiritual and social support resources are critical in reducing stress. Caregivers who reported stronger support systems, particularly in the form of spirituality and healthy lifestyles, experienced less stress, while those with higher physiological and psychological needs reported higher stress levels. Overall, the study emphasizes the need for comprehensive support systems that address not only the medical needs of pediatric cancer patients but also the emotional, psychological, and social needs of their caregivers. By understanding the multifaceted nature of caregiver stress and resource needs, healthcare providers can better tailor interventions to support caregivers throughout the treatment process, ultimately leading to improved care for both the patients and their families.

## Data Availability

The datasets generated and analyzed during the current study are not publicly available due to confidentiality agreements but are available upon reasonable request from the corresponding author.
