# Correction: Assessing caregiver stress and resource needs in pediatric cancer care

**DOI:** 10.1186/s12912-025-02695-9

**Published:** 2025-01-13

**Authors:** Shaimaa Mohamed Amin, Mahmoud Abdelwahab Khedr, Azza Medhat Aziz Mansy, Ahmed Hashem El-Monshed, Mohamed Gamal Noaman Malek, Ayman Mohamed El-Ashry

**Affiliations:** 1 Community Health Nursing, Faculty of Nursing, Damanhour University, Damanhour city, Egypt; 2 Psychiatric and Mental Health Nursing, Faculty of Nursing, Alexandria University, Alexandria, Egypt; 3 Department of Nursing, College of Applied Medical Sciences, Hafr Albatin University, Hafr Albatin, Saudi Arabia; 4 Psychiatric and mental health nursing faculty of nursing, Damanhour University, Damanhour, Egypt; 5 Department of Psychiatric and Mental Health Nursing, Faculty of Nursing-Mansoura University, Mansoura, Egypt; 6 Department of Nursing, College of Health and Sport Sciences, University of Bahrain, Manama, Bahrain; 7 Community Health Nursing, Faculty of Nursing, Minia University, Minia, Egypt; 8 Psychiatric and Mental Health Nursing, Department of Nursing, College of Applied Medical Sciences, Jouf University, Al Qurayyat, Saudi Arabia

**Correction:** ***BMC Nurs*** **23, 911 (2024)**

10.1186/s12912-024-02483-x

Following the publication of the original article [1], the author group identified an error in the affiliations for author Ayman Mohamed El-Ashry. The affiliation “Department of Nursing, College of Applied Medical Sciences, Hafr Albatin University, Hafr Albatin, Saudi Arabia” was incorrectly associated with author Ayman Mohamed El-Ashry and their affiliations should have been “Psychiatric and Mental Health Nursing, Faculty of Nursing, Alexandria University, Alexandria, Egypt” and “Psychiatric and Mental Health Nursing, Department of Nursing, College of Applied Medical Sciences, Jouf University, Al Qurayyat, Saudi Arabia”.

The original article [1] has been updated.
